# Feasibility of multiple-view myocardial perfusion MRI using radial simultaneous multi-slice acquisitions

**DOI:** 10.1371/journal.pone.0211738

**Published:** 2019-02-11

**Authors:** Ye Tian, Jason Mendes, Apoorva Pedgaonkar, Mark Ibrahim, Leif Jensen, Joyce D. Schroeder, Brent Wilson, Edward V. R. DiBella, Ganesh Adluru

**Affiliations:** 1 Utah Center for Advanced Imaging Research (UCAIR), Department of Radiology and Imaging Sciences, University of Utah, Salt Lake City, Utah, United States of America; 2 Department of Physics and Astronomy, University of Utah, Salt Lake City, Utah, United States of America; 3 Division of Cardiology, University of Utah, Salt Lake City, Utah, United States of America; Worcester Polytechnic Institute, UNITED STATES

## Abstract

**Purpose:**

Dynamic contrast enhanced MRI of the heart typically acquires 2–4 short-axis (SA) slices to detect and characterize coronary artery disease. This acquisition scheme is limited by incomplete coverage of the left ventricle. We studied the feasibility of using radial simultaneous multi-slice (SMS) technique to achieve SA, 2-chamber and/or 4-chamber long-axis (2CH LA and/or 4CH LA) coverage with and without electrocardiography (ECG) gating using a motion-robust reconstruction framework.

**Methods:**

12 subjects were scanned at rest and/or stress, free breathing, with or without ECG gating. Multiple sets of radial SMS k-space were acquired within each cardiac cycle, and each SMS set sampled 3 parallel slices that were either SA, 2CH LA, or 4CH LA slices. The radial data was interpolated onto Cartesian space using an SMS GRAPPA operator gridding method. Self-gating and respiratory states binning of the data were done. The binning information as well as a pixel tracking spatiotemporal constrained reconstruction method were applied to obtain motion-robust image reconstructions. Reconstructions with and without the pixel tracking method were compared for signal-to-noise ratio and contrast-to-noise ratio.

**Results:**

Full coverage of the heart (at least 3 SA and 3 LA slices) during the first pass of contrast at every heartbeat was achieved by using the radial SMS acquisition. The proposed pixel tracking reconstruction improves the average SNR and CNR by 21% and 30% respectively, and reduces temporal blurring for both gated and ungated acquisitions.

**Conclusion:**

Acquiring simultaneous multi-slice SA, 2CH LA and/or 4CH LA myocardial perfusion images in every heartbeat is feasible in both gated and ungated acquisitions. This can add confidence when detecting and characterizing coronary artery disease by revealing ischemia in different views, and by providing apical coverage that is improved relative to SA slices alone. The proposed pixel tracking framework improves the reconstruction while adding little computational cost.

## Background

Dynamic contrast enhanced MRI of the heart is becoming a more widespread tool to detect and characterize coronary artery disease. A standard protocol acquires 2–4 SA slices every heartbeat during the first pass of contrast agent, under stress and at rest. ECG gating and breath-holding are commonly employed to suppress motion. However, this acquisition suffers from limited coverage of the left ventricle myocardium, which can limit the sensitivity and confidence of detecting and quantifying ischemic regions. And when the ECG signal is poor, for example in patients with arrhythmias or patients who cannot hold their breath, the scans can be non-diagnostic.

Several approaches have been developed to address the issue of incomplete left ventricle coverage. By incorporating parallel imaging and compressed sensing with 2D SMS or 3D acquisitions [1–4], obtaining more SA slices per heartbeat is achievable. For example, a 2D radial SMS approach was used to acquire 12 SA slices [4], and a 3D technique was used to acquire 8 SA slices, but at a lower spatial resolution [2]. However, these studies were focused on increasing the number of SA slices rather than considering slices oriented differently such as LA slices. While previous studies that acquired LA slices were controversial on definitive advantages [5, 6], these studies were acquired at low spatial resolution and the systolic phase was not guaranteed. With an increased spatial resolution and systolic phase provided by self-gating, acquisition in different views may be helpful in corroborating ischemia detection and quantification of the ischemic burden. Seeing a perfusion deficit in two different views could increase confidence in reading the scan. Also a recent retrospective study using SPECT perfusion images pointed out that a conventional three slice acquisition is likely to fail to detect ischemia in the apical myocardium [7], and another study concluded that whole left ventricle coverage (6 SA slices) improves the accuracy in detection of coronary artery disease compared with acquiring 3 SA slices [8]. Acquiring full left ventricle coverage by SA slices and LA slices is of great interest; LA slices can probe part or all of the apical myocardium and full coverage of the 17 myocardial segments suggested by the American Heart Association [9] can be achieved.

In order to address the issues with ECG gating and breath holding, free breathing ungated acquisitions and motion compensation methods have been studied. In [10–12], retrospective self-gating was used by selecting a region of interest covering the heart to extract the cardiac signal and bin the data into near-systolic and near-diastolic cardiac phases. Motion compensation methods were incorporated into the reconstruction to achieve better image quality, by using rigid compensation [13], deformable compensation [14], pixel reordering [15] or patch-based approaches [4, 16].

The aim of this work is to study the feasibility of acquiring multiple views of myocardial perfusion under both gated and ungated conditions and to develop a fast and motion-robust reconstruction framework of radial SMS data. Radial controlled aliasing (CAIPI or CAIPIRINHA) SMS [17] combined with undersampling can capture several parallel slices simultaneously in a short time (~80ms), which not only snapshots the heart at a certain cardiac phase but also opens new possibilities in cardiac perfusion MRI. We used this technique to acquire multiple sets of SMS slices with different combinations of SA, 2CH LA and 4CH LA slices, under both gated and ungated conditions. A preliminary study has been done to acquire SA and 2CH LA slices with ECG gating [18]. For image reconstruction, the GRAPPA operator gridding (GROG) method [19, 20] was extended to grid radial SMS data to accelerate reconstruction. A motion compensated compressed sensing reconstruction framework was developed to resolve both cardiac and respiration motions, including pixel tracking temporal total variation and motion states binning.

## Methods

### Data acquisition

The University of Utah institutional review board approved this study. IRB #00100410. Written informed consent was obtained from all the participants in this study. The proposed acquisition uses a saturation recovery radial CAIPI turboFLASH sequence to acquire SMS k-space during the first-pass of contrast agent [3]. Multiple sets of SMS k-space were acquired within each cardiac cycle. A CAIPI phase modulation pattern of 0,0,0…; 0,2*π*/3, 4*π*/3…; 0, 4*π*/3, 2*π*/3… was applied continuously on golden angle spaced rays to achieve a multi-band factor of 3. The 90° saturation recovery (SR) pulse was optimized by using a 6-pulse train [21], and was applied before acquiring one or more SMS sets. 12 subjects were scanned—6 with gated and 6 with ungated acquisitions. For 9 subjects (5 gated and 4 ungated), the stress acquisition was performed ~60 seconds after regadenoson injection, then aminophylline was injected to reverse the effects of regadenoson and after ~10 min the rest acquisition was performed. 3 subjects (1 gated and 2 ungated) were scanned only at rest. 10 subjects were scanned on a 3T Prisma (Siemens) scanner and 2 were scanned on a 3T Skyra (Siemens) scanner. Siemens spine (32 channels) and body (18 channels) coils were used for acquisition. The channels were selected by the technologists. A total of 10 male subjects with a mean age of 62 years (±17 years) and 2 female subjects with a mean age of 71 years (±18 years) were scanned in this study. The subjects were informed of the possibility of joining the study by a cardiologist at the University of Utah and they may or may not have cardiomyopathies. All acquisitions share similar parameters: TR/TE = 2.7/1.6msec, FOV = 260mm, ~1.8x1.8x8mm pixel size, 30 rays/frame and flip angle = 12°. The contrast agent used in all scans was gadoteridol (ProHance, Bracco Diagnostics, Singen, Germany) at a dose of ~0.075mmol/kg for both rest and stress. For a few of the datasets, up to 72 rays/frame were acquired in order to study the impact of undersampling. A complete list of all datasets is shown in Table 1.

**Table 1:** List of all datasets. Gated scans acquired 9–18 slices per heartbeat, and ungated scans acquired 6–9 slices (~20–33 images per second) at 30 rays/frame. The SMS sets order also indicates the acquisition order after the saturation pulse.

Gated				
subject	Stress/rest	SMS sets	rays/frame	scanner
1	Stress	SA + 2CH + 4CH	30	Prisma
	Rest	SA x 2 + 2CH + 4CH	30	Prisma
2	Rest	SA x 4 + 2CH + 4CH	30	Skyra
3	Stress	2CH + SA x 2	51	Prisma
	Rest	2CH + SA x 2	51	Prisma
4	Stress	2CH + SA x 2	30	Prisma
	Rest	2CH + SA x 2	30	Prisma
5	Stress	2CH + SA x 2	30	Prisma
	Rest	2CH + SA x 2	30	Prisma
6	Stress	2CH + SA x 2	30	Prisma
	rest	2CH + SA x 2	30	Prisma

Ungated					
subject	Stress/rest	SMS sets	rays/frame	sequence	scanner
7	Stress	SA + 4CH	30	1	Prisma
	Rest	SA + 4CH	30	1	Prisma
8	Rest	SA + 4CH	72	1	Skyra
9	Rest	SA + 4CH	54	1	Prisma
10	Stress	LA + SA x 2	30	2	Prisma
	Rest	LA + SA x 2	30	2	Prisma
11	Stress	SA x 2 + LA	30	2	Prisma
	Rest	SA x 2 + LA	30	2	Prisma
12	Stress	LA + SA x 2	30	2	Prisma
	rest	LA + SA x 2	30	2	Prisma

For gated acquisition, 3–6 sets of SMS k-space were acquired after receiving an ECG trigger depending on the heartrate, and each SMS set sampled 3 parallel slices that were read out simultaneously after an SR pulse with ~80ms saturation recovering time (SRT), resulting in 9–18 slices per heartbeat. Subject 1 was scanned with 3 SA, 3 2CH LA and 3 4CH LA slices at stress, and at rest 3 more SA slices were acquired. Subject 2 was scanned only at rest with 12 SA, 3 2CH and 3 4CH LA slices. For subjects 3–6, 6 SA and 3 2CH LA slices were acquired at both stress and rest.

**Fig 1:**
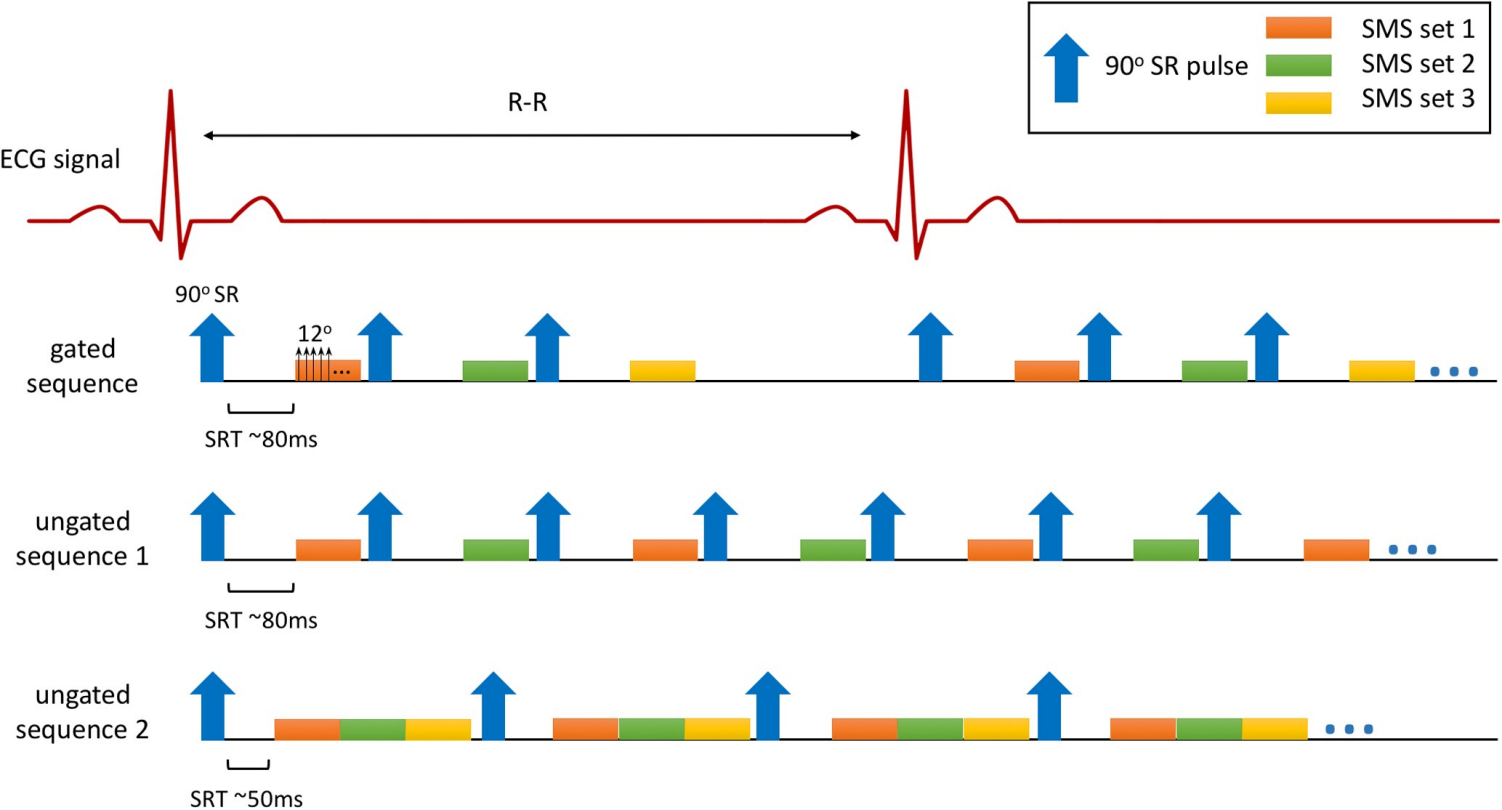
Pulse sequences used in this study. The gated sequence applies an SR pulse (composed of 6 pulses) after an ECG trigger followed by acquisition of an SMS set. 3–6 SMS sets were acquired depended on the heartrate. The ungated sequences continuously acquire 2 (sequence 1) or 3 (sequence 2) SMS sets, with one SR pulse applied for each SMS set (sequence 1) or one SR pulse applied for all SMS sets (sequence 2).

Two sequences were tested for ungated acquisitions. The first sequence was similar to the gated acquisitions in which each SR pulse was applied for each SMS set with SRT ~80ms, but without ECG trigging, and subjects 7–9 were scanned using this sequence for 3 SA and 3 4CH LA slices. The second sequence used only one SR followed by reading out of 3 SMS sets (2 SA and 1 2CH LA) with SRT ~50ms, and subjects 10–12 were scanned using this sequence. The ungated sequence ‘2’ provides more slice coverage than the ungated sequence ‘1’ (33 images/second over 20 images/second) but may cause more artifacts due to slice intersections. Fig 1 illustrates different pulse sequences used in this study.

10–20 proton density images were acquired with 30 rays/image at the beginning of each scan, using the same sequence but without saturation recovery pulse and with a flip angle of 2°. Late gadolinium enhanced (LGE) images were acquired ~10 minutes after the last contrast injection for all subjects, using a clinical free-breathing single shot inversion recovery sequence.

### Reconstruction

**Fig 2:**
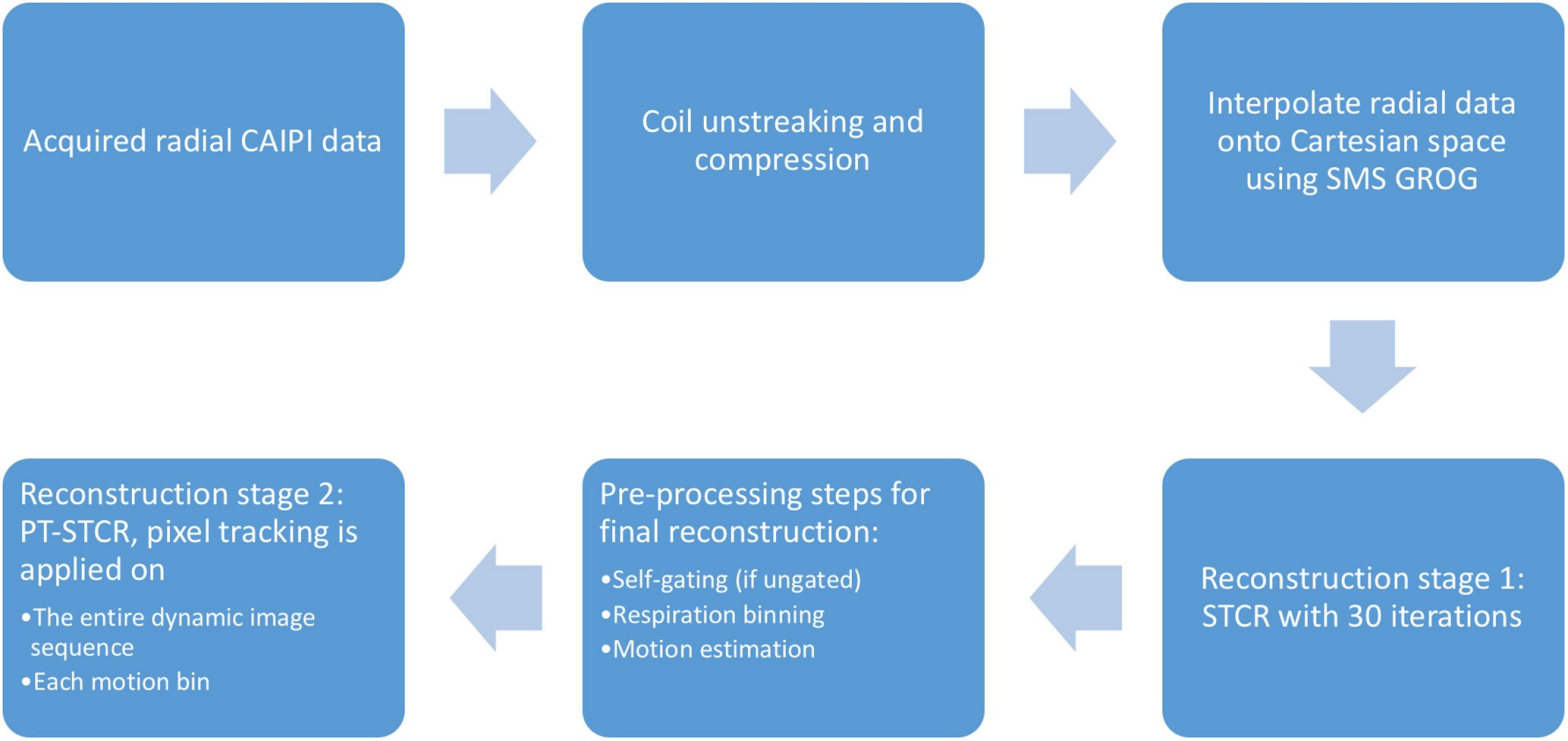
Flowchart describing the pre-processing and image reconstruction.

Fig 2 shows a flowchart of the image reconstruction process. The acquired multi-coil radial CAIPI data was first processed to suppress streaking artifact and compressed to have 8 virtual coils to speed up reconstruction. Then the radial data was interpolated onto Cartesian space using the SMS GROG method. The following reconstruction included two stages: In stage 1 the artifact-reduced reference images were reconstructed using spatiotemporal constrained reconstruction (STCR) [22]. By using these reference images, self-gating was done if ungated, the time frames were binned into different respiratory states and the inter-frame motion was estimated. In stage 2 the final reconstruction of motion-preserved images was performed by applying pixel tracking temporal total variation constraint on the entire dynamic image sequence (or near-systolic and near-diastolic phases if ungated) and each respiratory motion bin. All the processes were done automatically without any manual input. The following sections describe the reconstruction processes in more details.

#### Coil unstreaking and compression

Acquired radial k-space data were automatically processed to suppress streaking artifact using a coil unstreaking method proposed in [23]. This method calculates a streaking ratio based on the difference between an artifact-free image reconstructed from high number of rays (here 900 rays) and an image reconstructed from a low number of rays (here 90 rays) for each coil *c*:

$${streaking\;ratio}_{c}\;=\;\sum_{k}\frac{{∥10\;\times \;{Image}_{c,k,2\times FOV,90}-{Image}_{c,k,2\times FOV,900}∥}_{2}^{2}}{{∥{Image}_{c,k,1\times FOV,900}∥}_{2}^{2}}$$ (1)

The scale factor of 10 (900/90) in Eq (1) was used to compensate images reconstructed from different numbers of rays. The denominator and numerator use images of different field of view (FOV) to emphasize the signal from the region of interest. A summation on all simultaneously acquired slices *k* was performed to consider streaking from all slices. Coils that have a streaking ratio higher than 20 were scaled down by 20/streaking ratio_*c*_ in order to reduce their contribution in the final reconstructed images. After scaling of coils, principal component analysis (PCA) was performed along the coil dimension to compress the coil data by keeping only the first 8 principal components for reconstructions. This compresses the data to have 8 virtual coils and reduces computational demand without losing image quality [24].

#### Pre-reconstruction interpolation using SMS GROG

Pre-reconstruction interpolation methods are promising to speed up iterative reconstructions of non-Cartesian k-space since the computationally intensive interpolation steps when calculating projections and back projections can be avoided [20]. Several types of pre-interpolation methods have been proposed including bilinear interpolation[22], Toeplitz-based approach [25–27] and GRAPPA operator gridding or GROG [19, 28]. An iterative reconstruction framework of radial SMS data based on self-calibration GROG was developed to speed up reconstruction in this study.

**Fig 3:**
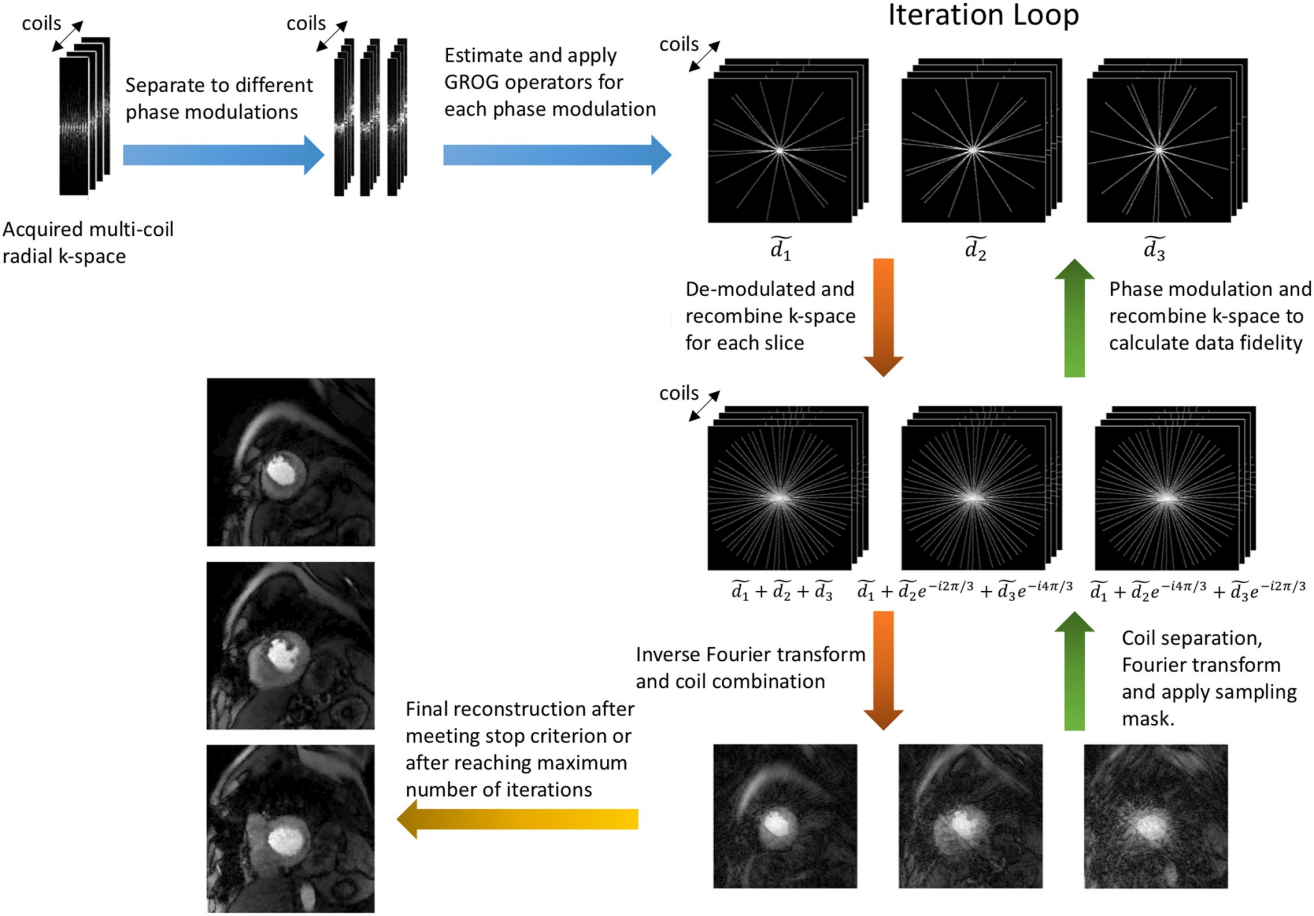
Iterative reconstruction using SMS GROG. The acquired radial k-space was first separated into different phase modulations, then within each phase modulation a set of GROG operators was trained and applied to interpolate the radial k-space onto a Cartesian grid. In the iteration loop, the Cartesian k-space was demodulated and modulated back and forth to calculate the data fidelity term.

The self-calibration GROG trains a set of GROG operators (*G*_*x*_, *G*_*y*_) using correlations among coil data, and then uses these operators to shift non-Cartesian k-space data onto a Cartesian grid [19, 20, 28]. Radial CAIPI applies different phase modulations on all simultaneously acquired slices [3, 17], which changes the data correlation among differently modulated k-space rays. However, within rays of the same phase modulation, the data correlation is the same. In order to grid CAIPI k-space data, different GROG operator sets were trained from different phase modulation k-space rays. In this study, multi-band factor of 3 was used for all acquisitions, thus for each acquisition 3 sets of GROG operators were trained from 3 sets of phase modulation k-space rays and applied to grid each set onto an undersampled Cartesian grid. The new Cartesian k-space instead of the acquired radial data were then used in data fidelity term as *d*. During iterations, the Cartesian k-space were demodulated and modulated back and forth to calculate data fidelity by using a matrix *Φ* as shown in Eqs (2, 3):

$${\;d}_{j}\;=\;\Phi_{jk}^{*}\tilde{d_{k}}$$ (2)

$$\Phi_{jk}\;=\left[\begin{array}{ccc} 1 & 1 & 1\\ 1 & e^{\frac{i2\pi }{3}} & e^{\frac{i4\pi }{3}}\\ 1 & e^{\frac{i4\pi }{3}} & e^{\frac{i2\pi }{3}} \end{array}\right]$$ (3)

where $\tilde{d_{k}}$ represents different phase modulation (represented by *k*) Cartesian k-space and *d*_*j*_ stands for k-space of different image slices *j*. These steps are incorporated into the sampling matrix such that *A* = *ΦMFS*, where *S* represents coil sensitivity maps, *F* is Fourier transform, *M* is a binary mask that simulates radial undersampling in Cartesian space and *Φ* is the phase modulation and recombination matrix. The initial estimate of *m* is calculated by applying the backward operator $A^{H}\;=\;S^{H}F^{-1}D\Phi_{jk}^{*}$ on the estimated Cartesian data $\tilde{d_{k}}$. Fig 3 illustrates the SMS GROG iterative reconstruction framework.

#### Reconstruction stage 1: STCR

In this reconstruction stage the STCR cost function [22] shown in Eq (4) was minimized:

$${∥Am-d∥}_{2}^{2}+\lambda_{s}{∥\sqrt{{\left(\nabla_{x}m\right)}^{2}+{\left(\nabla_{y}m\right)}^{2}+\epsilon }∥}_{1}+{\lambda_{t}∥\sqrt{{\left(\nabla_{t}m\right)}^{2}+\epsilon }∥}_{1}$$ (4)

where *d* is estimated multi-coil Cartesian k-space data (obtained by applying SMS GROG interpolation method on the radial SMS data), *A* = *ΦMFS* is the encoding matrix (forward projector) that includes phase modulation *Φ*, sampling mask *M*, Fourier transform *F* and coil-sensitivities *S*, *m* represents multi-slice dynamic images to be reconstructed, *λ*_*t*,*s*_ are temporal and spatial regularization parameters for the temporal and spatial total variation constraints respectively and *ϵ* is a small positive value used to avoid singularity. The back projector $A^{H}\;=\;S^{H}F^{-1}D\Phi_{jk}^{*}$ includes a density compensation matrix *D* calculated from radial sampling density which is used to accelerate convergence speed. We used 30 iterations (empirically determined) in the first stage to reduce the slice aliasing and undersampling artifacts. The weights were set at *λ*_*t*_ = 0.1*C*, *λ*_*s*_ = 0.03*C* where the *C* is the maximum signal intensity in the initial estimate of *m*.

#### Image-based self-gating and respiratory binning

Reference images from reconstruction stage 1 were used for self-gating and binning into respiratory states. The blood pools are ideal areas to extract cardiac signal where the intensities are low at pre-contrast frames and high during contrast arrival, thus two thresholds were used to segment the blood pools in each slice. First, a maximum intensity map was calculated, then the dynamic images were normalized by this map and a summation of the first 10 pre-contrast frames was done. The pixels with the lowest 20% intensities in the summation were selected as mask 1. Then from the maximum intensity map, pixels that had intensities larger than 0.4 of the maximum intensity was selected to be mask 2. Mask 1 and mask 2 were combined and filtered by a Gaussian filter to obtain the final segmentation. The selected areas from all simultaneously acquired slices were summed to be a 1D signal, then the signal was normalized to a 0–1 range by Eq (5):

$$normalized\;signal(t)\;=\;\frac{signal(t)-lower\;bound(t)}{upper\;bound(t)}$$ (5)

**Fig 4:**
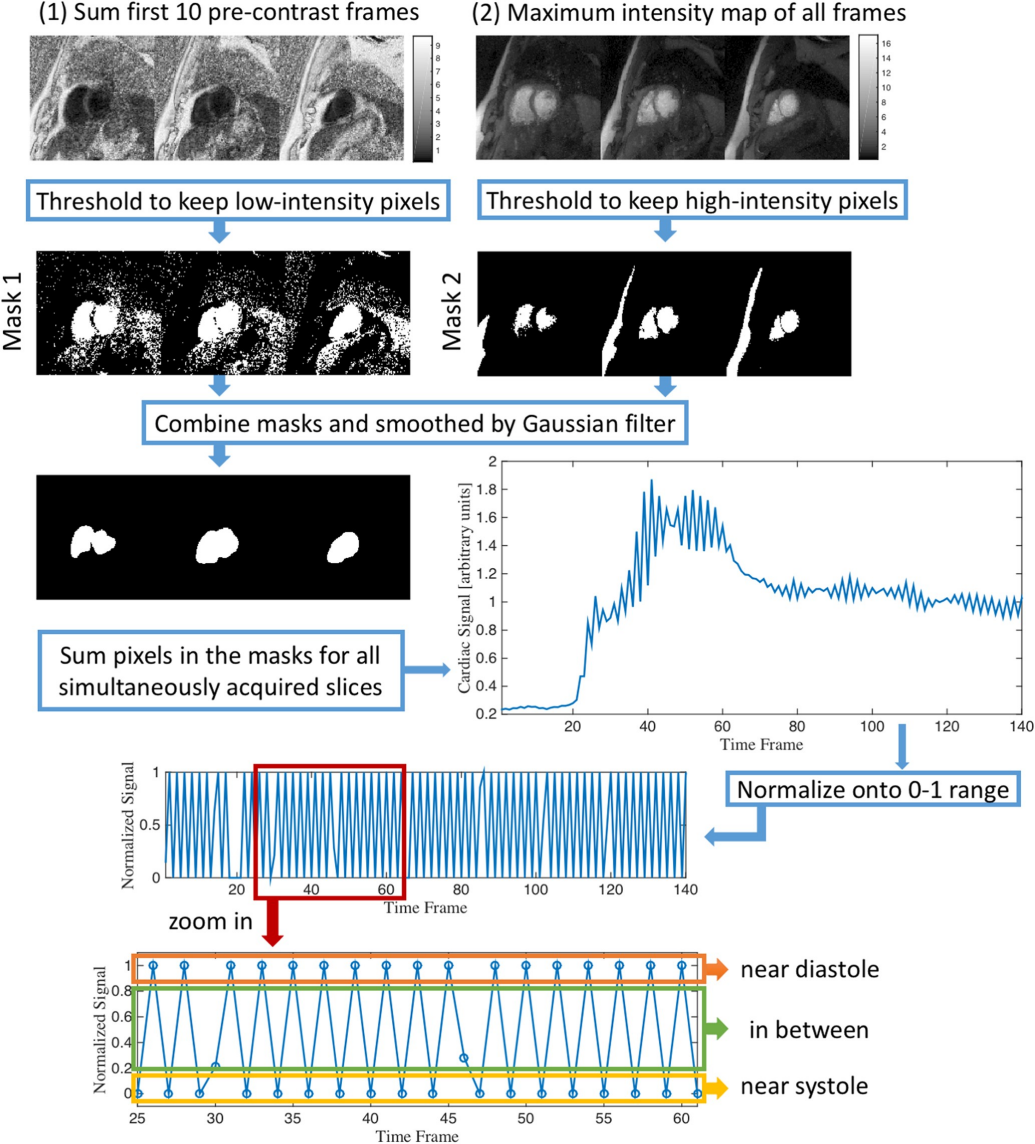
Self-gating steps for ungated acquisitions. Summation of the first 10 pre-contrast frames was threshold to keep low intensity areas to create mask 1, and maximum intensity map was threshold to keep high intensity areas to be mask 2. Then these two masks were combined and smoothed by a Gaussian filter to identify blood pools in all the simultaneously excited slices. The signals in the blood pool masks of all simultaneously acquired slices were summed together to give a 1D signal that was normalized to be between 0 and 1. Near-systolic and near-diastolic frames were selected from the normalized signal.

The upper (lower) bound was found by first linearly interpolating the local maxima (minima) of the signal onto all time points, and then fine tuning the bound to be higher (lower) than the signal at all time points. Finally, all time frames were binned into 3 cardiac phases (a near-systolic, a near-diastolic and an in-between phase) from the normalized signal. Fig 4 shows this pipeline.

**Fig 5:**
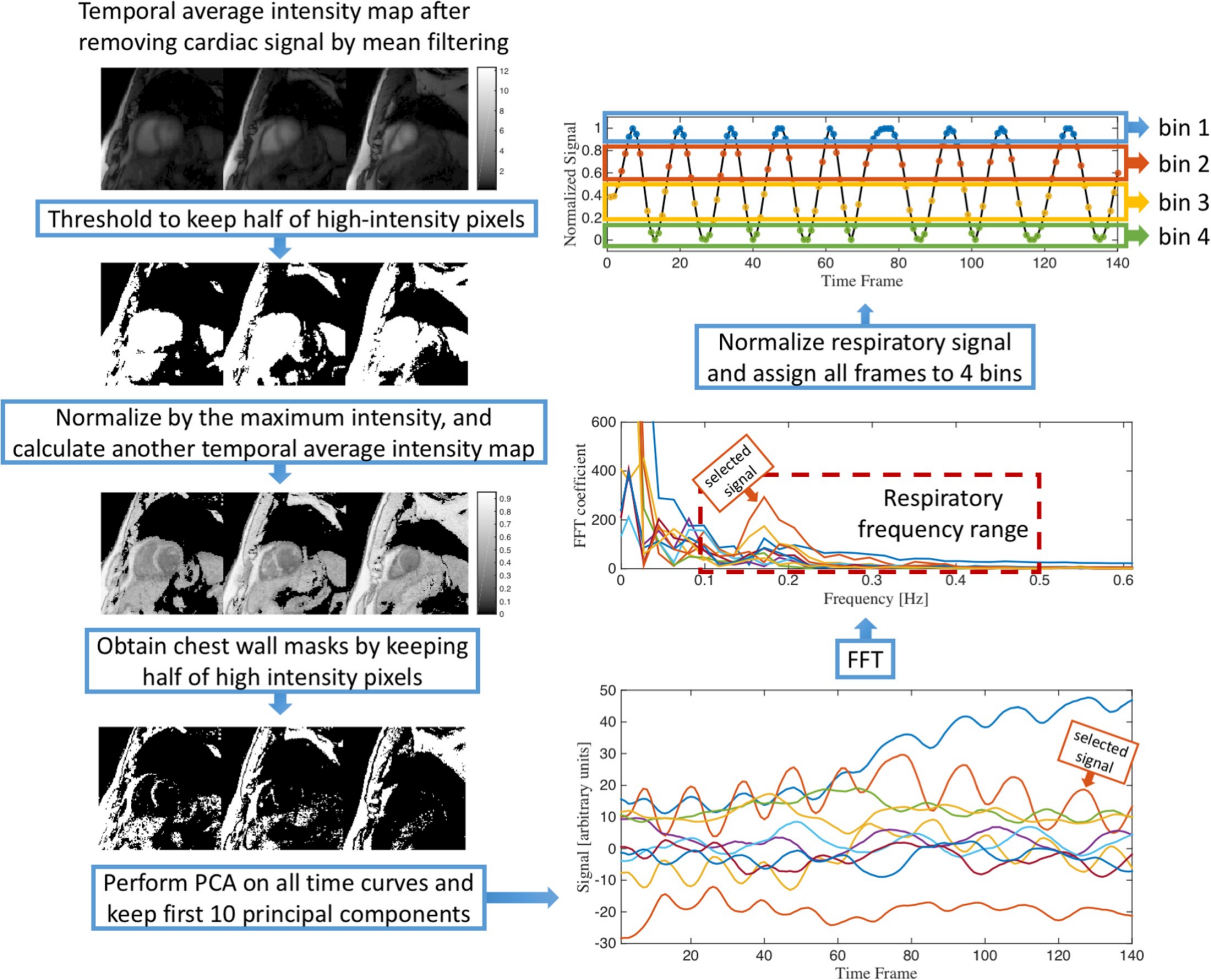
Illustration of the respiratory binning process. An average intensity map was first calculated by averaging pixel intensities along the time dimension using mean-filter-smoothed dynamic images. From this map, the half of the pixels with the highest intensity were kept and then normalized by each pixel’s highest intensity along time. Another average intensity map was calculated using the normalized data and half of the highest intensity pixels in this map were selected in order to mainly preserve the chest wall signal. PCA was applied on the selected pixels. The PCA component that has highest intensity in frequency range 0.1–0.5 Hz (within the first 10 principal components) was selected and normalized to be the respiratory gating signal. All time frames were binned into 4 respiratory bins using the normalized signal.

The chest wall is a good indicator of respiratory motion, thus we designed thresholding methods to segment the chest wall as follows. The dynamic images were first smoothed by a mean filter of width 3 to remove the overlapping cardiac signal. Then, half of the pixels with the lowest intensities in the average intensity map were dropped so that the low signal areas such as lung or background were excluded. Next, to remove the blood pool areas, the images were normalized by a maximum intensity map, and an average intensity map was calculated. The blood pools then had low intensities and were removed by dropping half of the pixels with the lowest intensities. All of the remaining pixel time curves were sorted together and PCA was applied along the pixel dimension, then the component that had the highest signal at frequency 0.1–0.5 Hz was selected and normalized into a 0–1 range to be the respiratory signal. From this respiratory signal, all time frames were binned into 4 respiratory states that all have similar number of frames. Fig 5 shows a flowchart of this process.

#### Pixel tracking temporal total variation

Temporal total variation constraints have been applied to reconstruct multiple types of dynamic MRI data successfully [22, 29, 30]. Pixel intensity time curves are assumed to be piece-wise smooth, or the first order derivative along time dimension is assumed to be sparse. Such an assumption breaks down when inter-frame motion is present since motion misaligns pixels representing the same feature along the time dimension. To mitigate this effect, we propose a unique approach to estimate the motion trajectory for each pixel using deformable registration from the pre-reconstructed reference images, and then calculate the temporal total variation along the estimated motion tracks.

When a gradient based algorithm is employed to solve the cost function in (4), seeking an numerical update for the temporal term of each time frame *m*_*i*_ requires its neighboring time frames *m*_*i*−1_ and *m*_*i*+1_:

$$m_{i}^{update}\;=\;\frac{m_{i+1}-m_{i}}{\sqrt{{\left(m_{i+1}-m_{i}\right)}^{2}+\epsilon }}-\frac{m_{i}-m_{i-1}}{\sqrt{{\left(m_{i}-m_{i-1}\right)}^{2}+\epsilon }}$$ (6)

In order to calculate this update term along the motion track of each pixel, we reorder the pixels in the *i* − 1th and *i* + 1th frames using reordering matrices *P*_*i*+1→*i*_ and *P*_*i*−1→*i*_:

$$m_{i}^{update}\;=\;\frac{P_{i+1\to i}m_{i+1}-m_{i}}{\sqrt{{\left(P_{i+1\to i}m_{i+1}-m_{i}\right)}^{2}+\epsilon }}-\frac{m_{i}-P_{i-1\to i}m_{i-1}}{\sqrt{{\left(m_{i}-P_{i-1\to i}m_{i-1}\right)}^{2}+\epsilon }}$$ (7)

**Fig 6:**
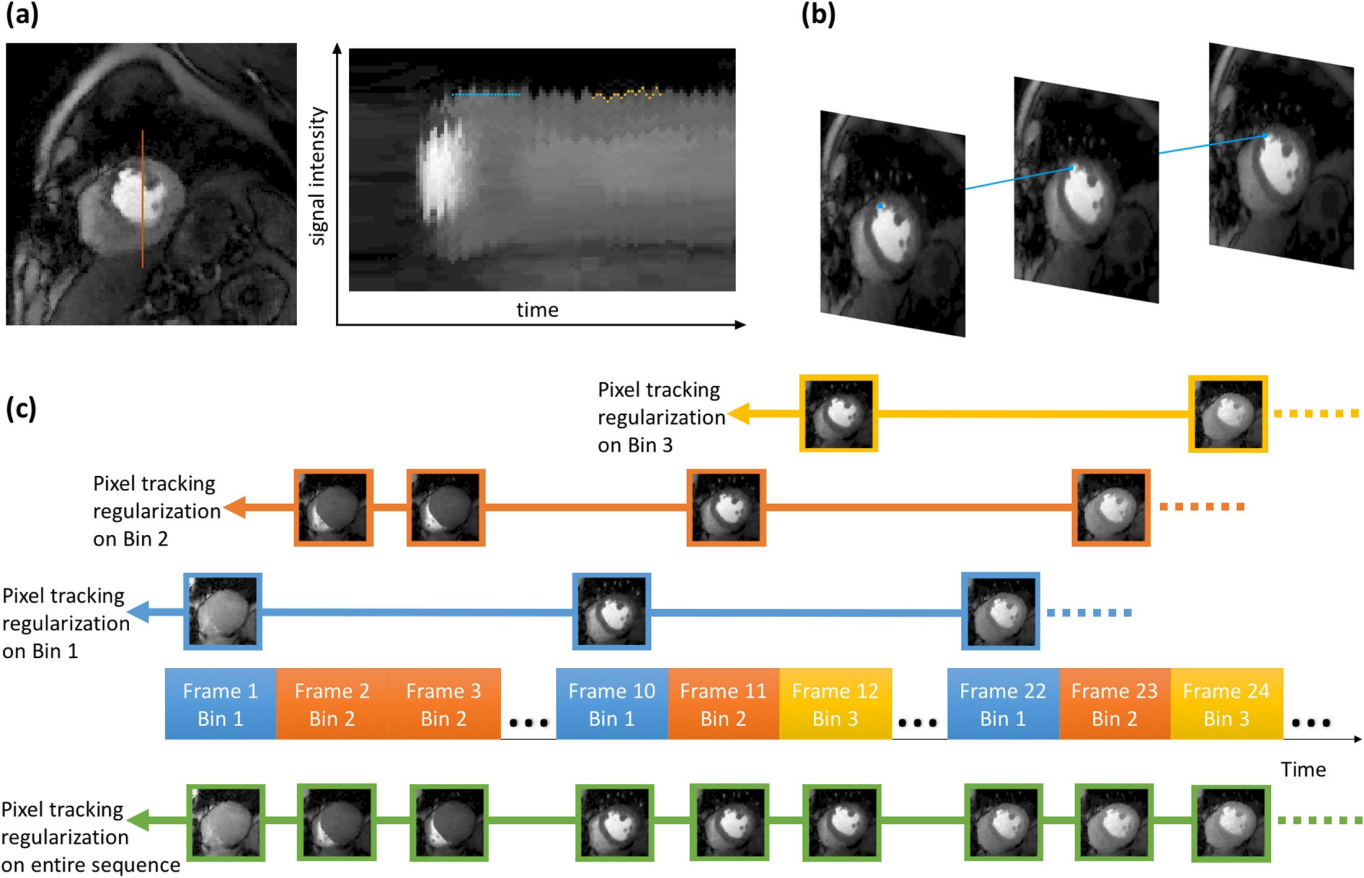
Illustration of pixel tracking temporal regularization. (a) The right image shows the signal intensity line profile over time of the red line in the left image. Standard temporal total variation calculates total variation along the blue line, however pixel tracking temporal total variation calculates total variation along the yellow line. (b) The blue line shows estimated motion track of one pixel at the edge of blood pool. (c) Pixel tracking regularization is applied on both the entire image sequence (shown below the time axis), as well as each motion bin (shown above the time axis).

Similar to the MASTeR approach [31], two sets of motion maps were estimated: forward motion maps that register frame *m*_*i*−1_ to time frame *m*_*i*_ and backward maps. The registration was performed using large deformation kinematics with a greedy algorithm [32], and an intensity correction map was estimated to help registration [33]. Then the reordering matrix *P* can be produced from these maps that spatially align each pixel along its time track. Fig 6(a) and 6(b) shows the estimated pixel track in 2D and 3D cases.

#### Reconstruction stage 2: PT-STCR

Then the following cost function was minimized for stage 2 reconstruction:

$${∥Am-d∥}_{2}^{2}+\lambda_{s}{∥\sqrt{{\left(\nabla_{x}m\right)}^{2}+{\left(\nabla_{y}m\right)}^{2}+\epsilon }∥}_{1}+{\lambda_{t}∥\sqrt{{\left(\nabla_{t}Pm\right)}^{2}+\epsilon }∥}_{1}+\lambda_{t}\sum_{n\;=\;1}^{nbins}{∥\sqrt{{\left(\nabla_{t}P_{n}m_{n}\right)}^{2}+\epsilon }∥}_{1}$$ (8)

where *P*, *P*_*n*_ are the reordering matrices for the entire image sequence and for each motion bin *n*, respectively. For ungated acquisitions, *m* stands for near-systolic or near-diastolic frames after self-gating since the two phases were reconstructed separately, and the in-between phase was excluded in this stage. As shown in Eq (8), the pixel tracking regularization was applied twice. The third term applies the pixel tracking on all the time frames, while the forth term applies the pixel tracking on each motion bin *n*, and 4 respiratory bins were used for all datasets in this study. This reconstruction scheme (later referred as PT-STCR) is shown in Fig 6(c). The reference images from stage 1 were used as initial estimation for stage 2 reconstruction. In the reconstruction stage 2, the maximum iteration number was set at 100 and the iteration were stopped when: (1) backtracking line searching finds a step size smaller than 1e^-4^ or (2) the cost functional changes less than 1e^-4^. The weights were set at *λ*_*t*_ = 0.15*C*, *λ*_*s*_ = 0.03*C* where the *C* stands for highest intensity in the initial estimate of *m*. A lower temporal constraint weight (*λ*_*t*_ = 0.1*C*) was used in the first stage to preserve more inter-frame motion when reconstructing the reference images.

#### Evaluation of PT-STCR

For both gated and ungated acquisitions, STCR reconstructions without pixel tracking were performed and compared with the PT-STCR. For ungated acquisitions, the STCR was performed on near-systolic and near-diastolic phases separately after the self-gating, and the self-gating was done using the previously stated method after 30 iterations. PT-STCR and STCR reconstructions used the same spatial regularization parameter *λ*_*s*_ = 0.03 *C* and for temporal regularization $\lambda_{t}^{PT-STCR}\;=\;0.15C$ and $\lambda_{t}^{STCR}\;=\;0.3C$ was used. These parameters were chosen empirically by visual evaluation. We picked 3 SA, 1 2CH LA and/or 1 4CH LA slices that have the most clinical value from all acquired slices of each scan to calculate the apparent signal-to-noise ratio (SNR) and the apparent contrast-to-noise ratio (CNR). One time frame in each slice near the peak-enhancement of myocardium was selected and segmented based on the 17-segment model by American Heart Association [9]. Then the same segmentation was applied on both PT-STCR and STCR images to calculate the SNR and CNR using Eq (9):

$${SNR}_{s}\;=\;\frac{\mu_{s}^{MYO}}{\sigma_{s}^{MYO}},\;{CNR}_{s}\;=\;\frac{\left|\mu^{LV}-\mu_{s}^{MYO}\right|}{\sigma_{s}^{MYO}}$$ (9)

where $\mu_{s}^{MYO}$ and $\sigma_{s}^{MYO}$ are the mean and standard deviation of each myocardial segment *s*, and *μ*^LV^ is the mean of the left ventricle.

The MATLAB (The MathWorks, Natick, MA) codes used in this study along with multi-view raw SMS datasets from representative ECG-gated and ungated acquisitions can be found at https://github.com/gadluru/Multiview-myocardial-perfusion-with-radial-SMS.

## Results

### Gated acquisition

**Fig 7:**
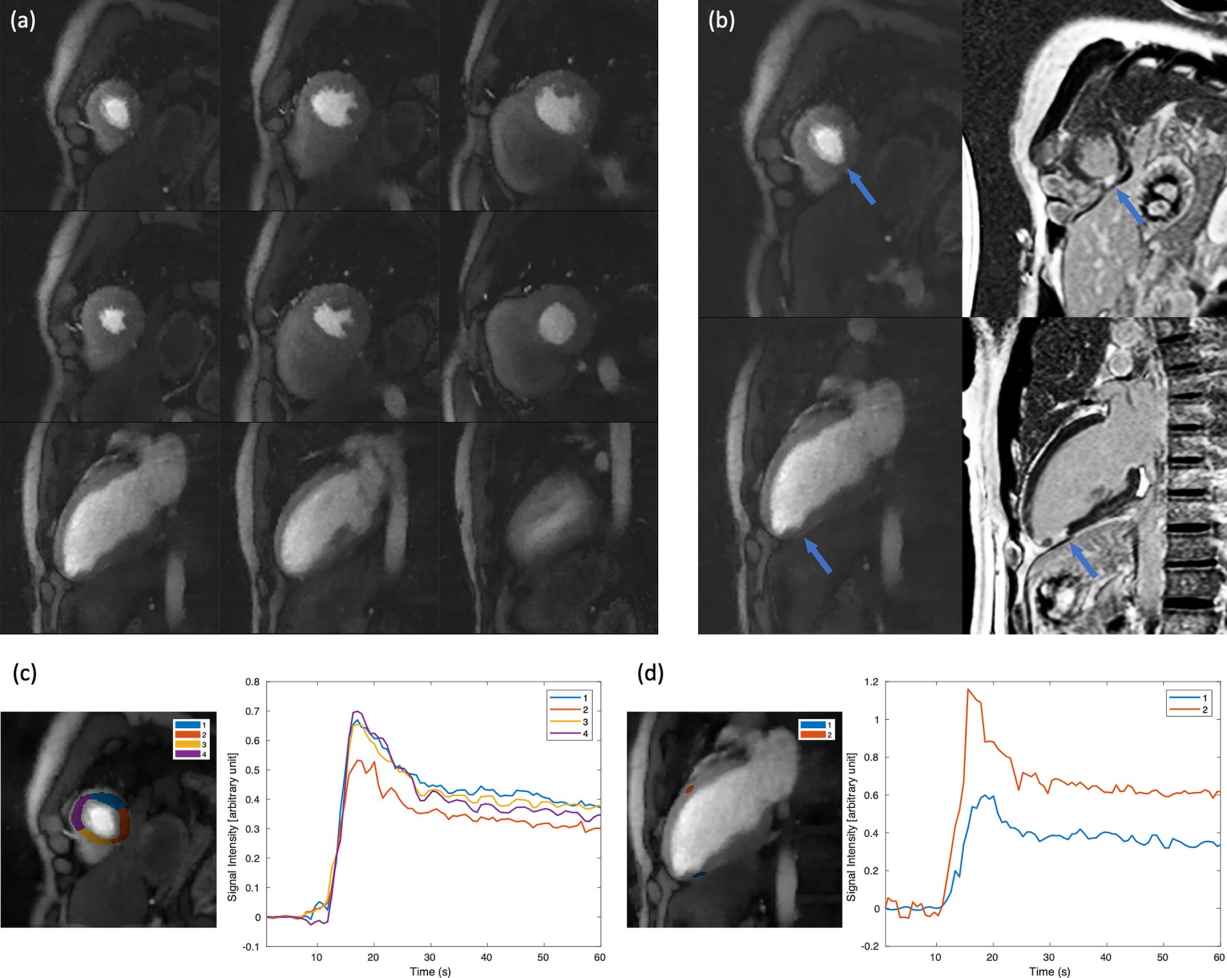
Results from an ECG-gated stress acquisition with a small perfusion deficit (subject 6). 6 SA slices and 3 2CH LA slices were acquired within each heartbeat. (a) shows all slices near peak myocardial enhancement. (b) shows a SA slice and a 2CH LA slice closely matching the corresponding LGE images are shown. A small area of deficit can be seen on both slices pointed by the arrows. This is more clearly seen in the time curves shown in (c), (d) for the corresponding ROI. Model-based registration [34] was performed before image segmentation. The time curves were normalized by proton density images and the pre-contrast baseline signal was subtracted.

**Fig 8:**
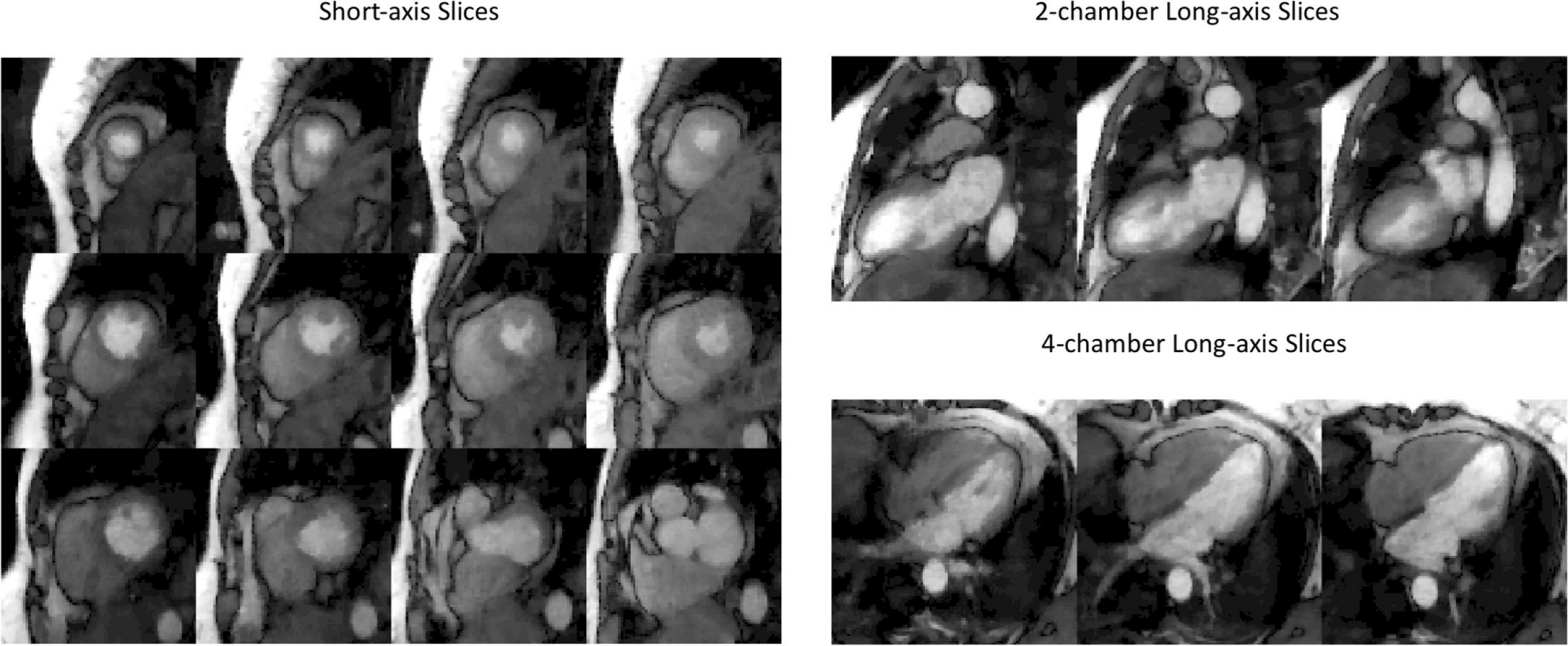
Illustration of an 18 slice acquisition at rest in subject 2. 12 SA, 3 2CH LA and 3 4CH LA slices were all acquired within one heartbeat for every heartbeat. For the SA slices, each column was acquired as an SMS set, and the 2CH LA slices or 4CH LA slices were each acquired as separate SMS set.

Fig 7 illustrates selected images of subject 6, where a small perfusion deficit is seen on both long-axis and short-axis images and matches the enhancement on the LGE images. Fig 7c and 7d show the signal intensity time curves for the ROIs from the short-axis and long-axis slices respectively. Ischemic regions have reduced signal enhancement compared to the normal tissues. In the rest acquisition of subject 2 when heart rate was low (~60/s), we were able to acquire 6 SMS sets with 12 SA, 3 2CH LA and 3 4CH LA slices, which is shown in Fig 8. Corresponding video (S1 Video) can be found in supplementary files.

### Ungated acquisition

**Fig 9:**
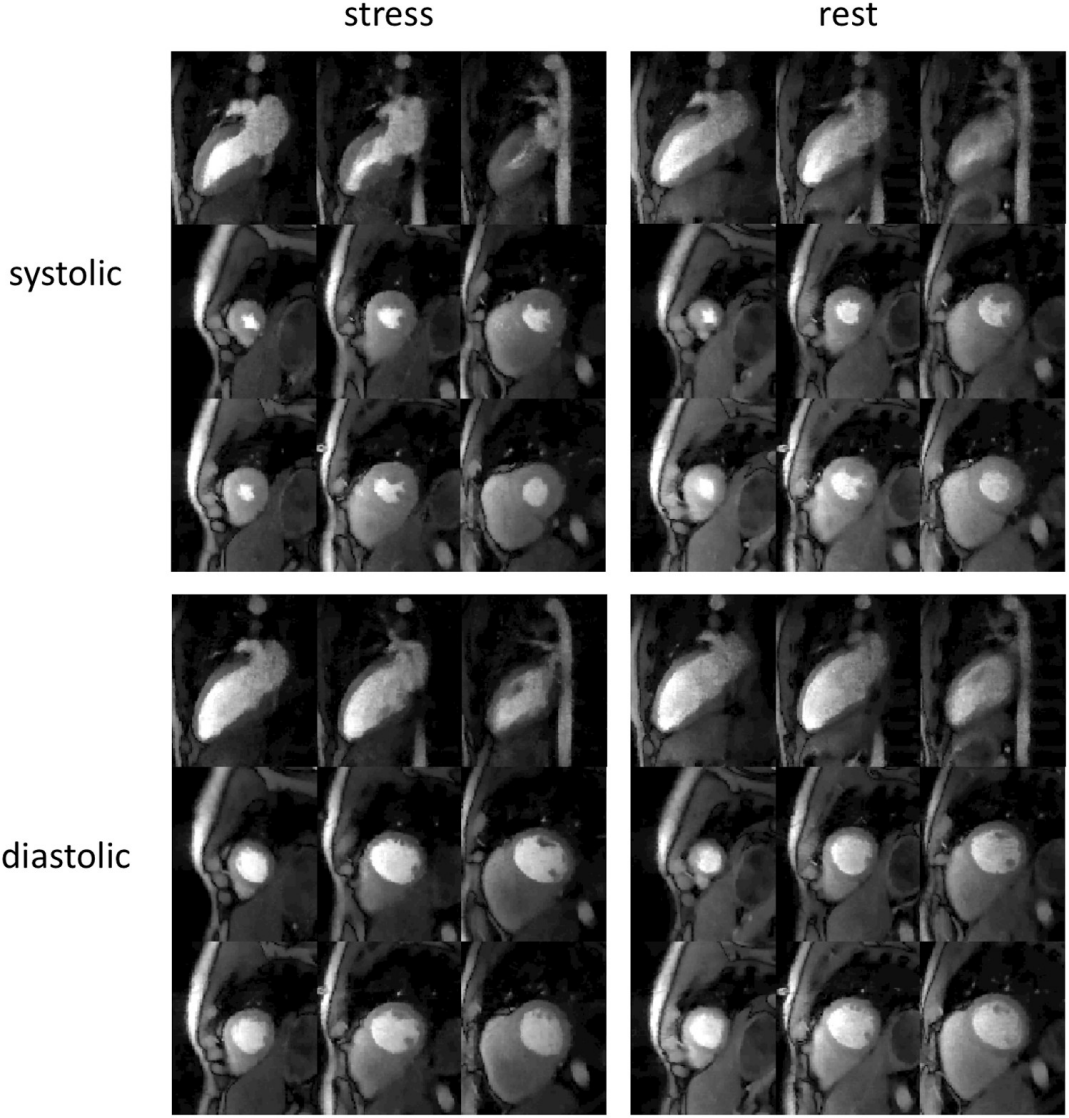
Results from an ungated acquisition (subject 10). Stress and rest images for systolic and diastolic phases are shown. In each image three slices in a row were acquired simultaneously. Time frames were picked to show peak myocardial enhancement.

Fig 9 shows ungated acquisition of subject 9, with 3 SA and 3 2CH slices, at both stress and rest for near-systolic and near-diastolic cardiac phases. The self-gating method was able to bin the time frames into near-systolic and near-diastolic phases. Corresponding video (S2 Video) is provided in supplementary files.

**Fig 10:**
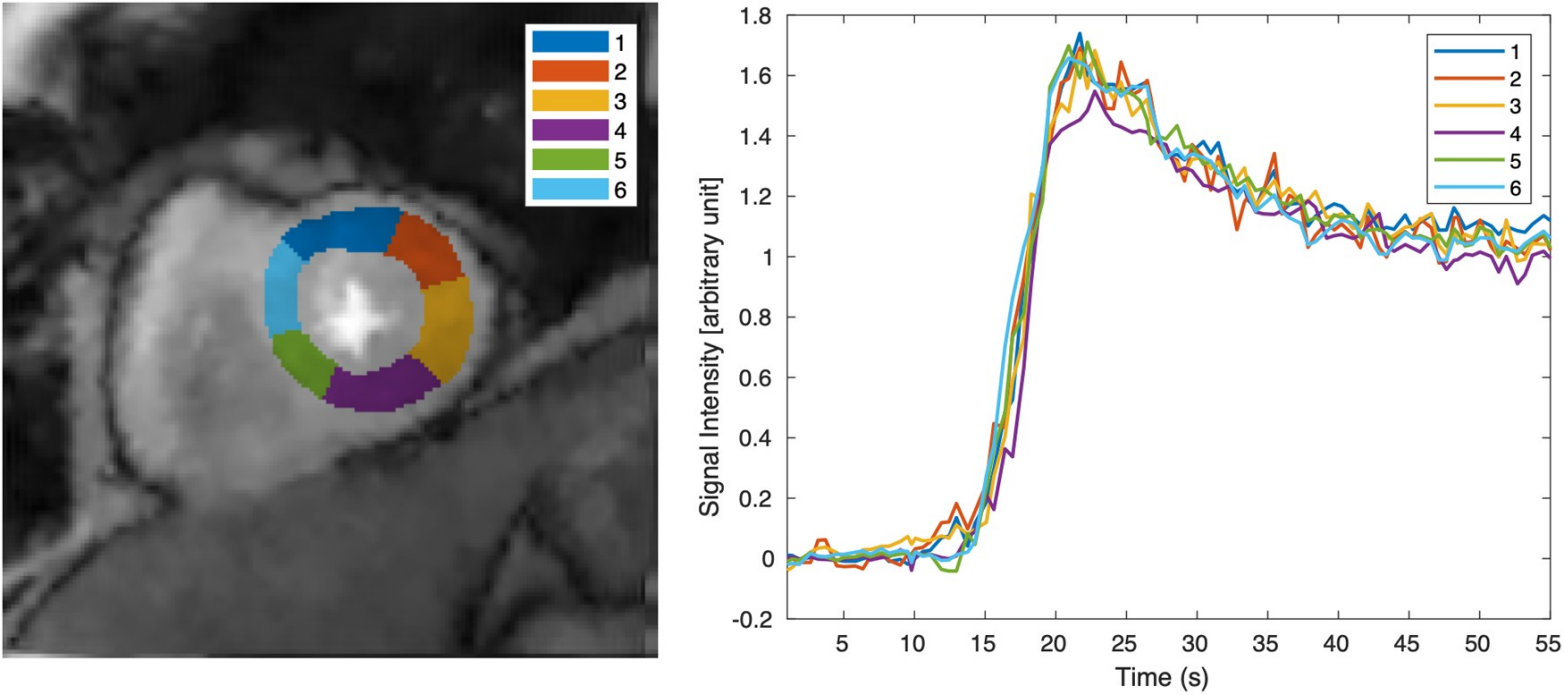
Tissue time intensity curves from an ungated stress acquisition (subject 12). The left image shows the selected slice with six cardiac segments. The right image shows the tissue intensity curves of the corresponding segments.

Fig 10 shows signal intensity time curves in a mid-ventricular SA slice from subject 12 acquired at stress. Systolic images were used. The images underwent model based registration [34] and six regions of myocardium were manually segmented according to the standardized approach [9]. The curves were normalized by the proton density images and the pre-contrast baseline signal was subtracted.

**Fig 11:**
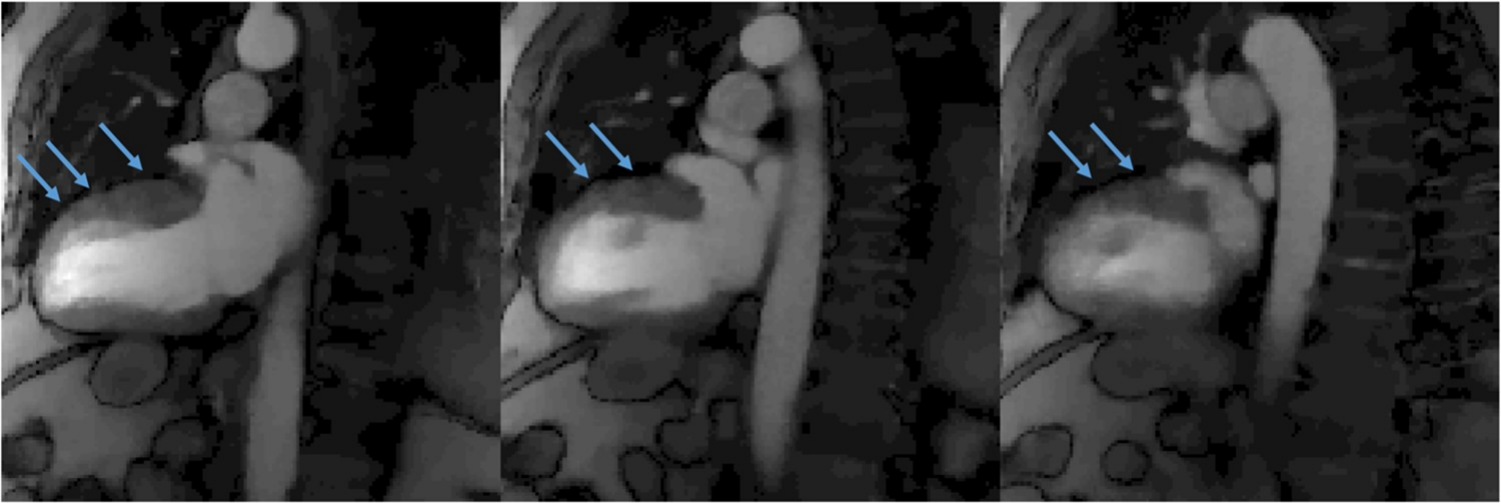
Dark band artifacts using overlapping slice positions. Faint dark bands (pointed by blue arrows) can be seen on all of the simultaneously acquired 2CH LA slices at slice intersections with previously acquire SA slices. Images were from subject 11.

For the two ungated sequences we tested, the ungated sequence ‘2’ (as illustrated in Fig 1) achieves more slice coverage than sequence ‘1’, but it may lead to more artifacts when the sequentially acquired slice groups intersect. We observed that when LA slices were acquired after the acquisition of SA slices (subject 11), faint dark bands appear (shown in Fig 11). This is because of the slice intersections with previously acquired SA slices that have experienced more readout pulses. Even though we did not find increased artifact in the LA-then-SA approach (subject 10 and 12, as shown in Fig 9 for subject 10), we propose not to use one SR preparation pulse to sequentially acquire slices that have intersections. The gated sequence and ungated sequence 1 (as shown in Fig 1) used a SR pulse for each SMS set did not have this artifact.

### Evaluation of PT-STCR

**Fig 12:**
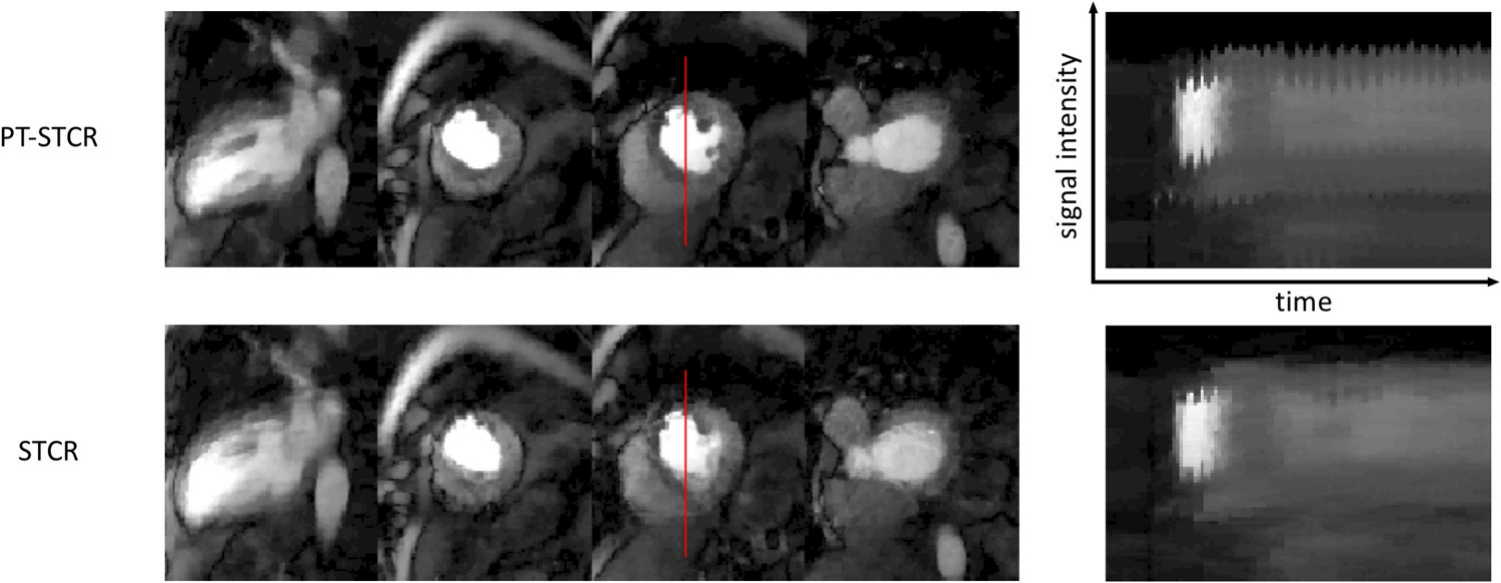
Comparison of PT-STCR and STCR in subject 4. 3 SA and 1 2CH LA slices were used in the SNR/CNR analysis are shown (9 slices were acquired). Vertical red lines on the images are used for comparison of signal intensity line profiles over time. PT-STCR reconstruction preserves the myocardial border better than STCR and has fewer artifacts. Images showing the signal intensity line profiles over time show that the PT-STCR reconstruction is sharper than the STCR reconstruction.

**Fig 13:**
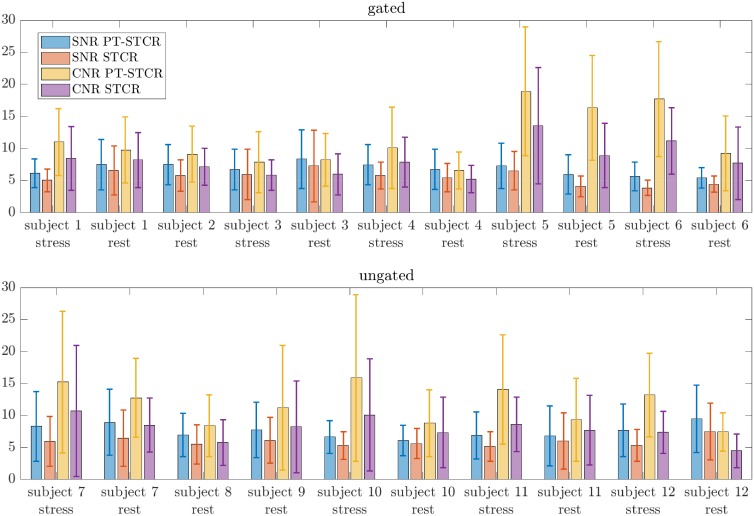
SNR and CNR results. The results shown here are averaged across all segments of SA, 2CH LA and/or 4CH LA slices from one Gadolinium injection. For ungated acquisitions, near-systolic and near-diastolic images were included in averaging. PT-STCR reconstructions have higher SNR and CNR than STCR reconstructions overall.

PT-STCR reconstructions resulted an average 21% more SNR and 30% more CNR compared with STCR reconstructions. Paired t-test resulted in *p* < 10^−14^ for SNR and *p* < 10^−20^ for CNR, indicating statistically significant differences between PT-STCR and STCR in terms of SNR and CNR. A comparison of STCR and PT-STCR reconstructions is shown in Fig 12. SNR and CNR results for all the subjects are illustrated in Fig 13.

## Discussion

In this study we tested the feasibility of acquiring different views of myocardium perfusion by using radial SMS. We were able to achieve full coverage of 17 myocardial segments suggested by American Heart Association [9] during first-pass perfusion including the apical segment typically missed by traditional 3 SA slices coverage. Compared with previous studies on acquiring LA slices that acquired 3 SA slices and 0–2 LA slices at a spatial resolution 2.7 x 3.6 mm to 3.1 x 4.1 mm [5], or acquired 4–7 LA slices at a spatial resolution 3.5 x 1.9 mm [6], the unique acquisition framework proposed here is able to achieve high resolution (1.8 x 1.8 mm) multi-view coverage in patients with high heartrate (up to 120 bpm) and also in patients with poor ECG gating, where both SA and LA slices at systolic and diastolic phases are guaranteed by ungated acquisitions. These advantages can increase the confidence when reading the scans. As well, the acquired LA views were matched with other acquisitions like cine, T1 maps and LGE images during the same scan, which collectively can provide a more confident detection of cardiac diseases.

**Fig 14:**
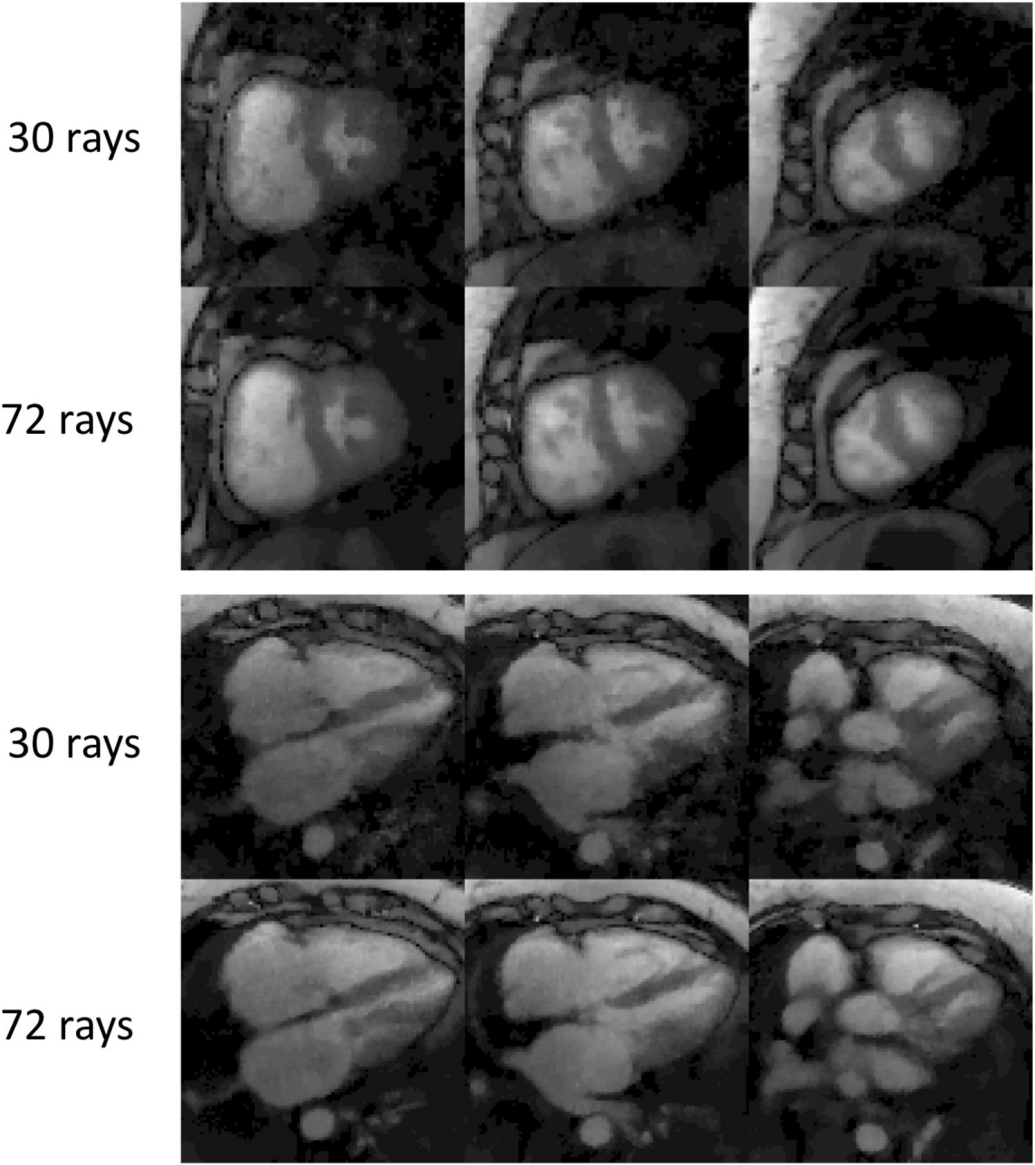
Comparison of undersampled reconstructions in a 72 rays/frame dataset (subject 8). Simulated undersampling was done by keeping only the first 30 rays. The 72 rays reconstruction is slightly better than the 30 rays reconstruction, however both reconstructions have high quality and contrast.

For most of the datasets in this study, 30 rays/frame were acquired. Acquiring more rays may result in better image quality, with the trade-off of increased acquisition time and increased saturation recovery effect on the image contrast. To study these trade-offs, in some of the datasets as listed in Table 1, we acquired more rays/frame and compared reconstructions obtained using first 30 rays of each time frame to those obtained using all rays. As can be seen on Fig 14 (subject 8), the 72 rays reconstruction has slightly better image quality than the 30 rays reconstruction, but both reconstructions have high quality and contrast. Since acquiring more rays may not lead to more clinical value, we acquire 30rays/frame for multiband 3 radial SMS cardiac perfusion studies.

An improved self-gating method is used in this study that can successfully bin acquired time frames into near-systolic and near-diastolic phases. In previous methods [10, 12], a rectangular region around the heart was selected after motion correction and sum of pixels in the region was used as cardiac signal, and all times frames were binned into two cardiac phases (near-systolic and near-diastolic). In [11], an automatic region of interest selection was performed by using a standard deviation map and finding a circular region that covered the heart, and only near-systolic frames were selected. The method used here automatically selected the left and right ventricles from the pre-contrast frames and the maximum intensity map. Instead of using geometric masks, the natural shapes of blood pools were preserved which is advantageous in indicating the cardiac signal. Since the ungated acquisition acquired data across all cardiac phases, some of the frames are acquired outside the systolic and diastolic phases. Consequently, all time frames were binned into three cardiac phases, a near-systolic, a near-diastolic and an in-between phase. The in-between phase was not included into reconstruction stage 2 to prevent artifacts in the systolic and diastolic data sets. By normalizing the cardiac and respiratory signal onto a 0–1 range, the self-gating and respiratory binning were efficiently done by thresholding. Also this normalization can detect small variations on the signal which were not local maxima or minima, especially for the cardiac signal (e.g. frame 60–70 in Fig 4 before normalization), which could be binned into the wrong phases.

The reordering operation *P* in PT-STCR is equivalent to applying a nearest neighbor interpolation using the estimated deformation map. The advantage of using nearest neighbor interpolation is that no pixel intensities in the neighboring time frames are changed when calculating total variation, and that the operation is faster compared to applying a more complex interpolation. The reconstruction speed of PT-STCR is comparable with STCR except for the extra registration steps. Though not shown here, we tested using bilinear interpolation instead of nearest neighbor, and found no visible difference in final reconstructed image quality. We note that the reordering used in PT-STCR differs from previously proposed reordering methods such as [15], which performed reordering on both spatial and temporal directions, and based the reordering on pixel intensities rather than deformation maps.

By binning all acquired time frames into different respiratory bins, we were able to apply the pixel tracking regularization on each bin (fourth term in Eq (8)) while applying pixel tracking on the entire image sequence (third term in Eq (8)). Grouping of frames having similar motion states together increases the temporal sparsity and leads to better motion preservation. Using different constraint weights for these two regularizations terms in Eq (8) may result in better reconstruction. We found that using the same constraint weights was robust across all 12 subjects in this study and no fine tuning was necessary. The robustness of the constraint weights should attribute to the pixel tracking that by aligning pixels before calculating temporal total variation, adjacent frames are motion-free such that the weights should be general when acquiring data in similar circumstances.

SA, 2CH LA and 4CH LA slices may be combined into an isotropic whole heart 4D image as has been done in cardiac cine MRI [35]. That work acquired 10–14 slices of each view to cover the whole heart; afterward all images were registered and a geometric super resolution reconstruction was applied to reconstruct a whole heart cine image with 1.4x1.4x1.4 mm resolution. The proposed acquisition here can provide multiple views of the heart during perfusion, which may make whole heart isotropic first-pass perfusion MRI possible. Also a simultaneous orthogonal slices technique [36] could possibly benefit the multiple-view acquisition by acquiring 2CH LA and 4CH LA slices simultaneously, but with unavoidable artifact at the slice intersections.

## Conclusion

Our results suggest that acquiring SA, 2CH LA and/or 4CH LA perfusion images using radial SMS technique is feasible in both gated and ungated myocardial perfusion dynamic contrast enhanced imaging. Combined SA and LA coverage may add confidence when detecting and characterizing coronary artery disease by revealing perfusion deficits in different views, and providing apical coverage that is improved relative to short axis slices alone. The self-gating and respiratory binning methods can separate acquired time frames into near-systolic and near-diastolic phases and different respiratory states. The proposed reconstruction framework using SMS GROG pre-interpolation and a motion robust iterative PT-STCR reconstruction improves the reconstruction of undersampled cardiac perfusion MRI while adding little computational cost.

## Supporting information

S1 Video Video corresponds to Fig 8. Video shows a gated acquisition when the patient had low heart rate (~60/min). 9 SA, 3 2CH LA and 3 4CH LA slices were acquired during each cardiac cycle.
(MOV)

S2 Video Video corresponds to Fig 9. Video shows an ungated acquisition case. 6 SA and 3 2CH LA slices were acquired during each cardiac cycle, and both self-gated systolic and diastolic images are shown.
(MOV)
